# Rodent Models of Alzheimer’s Disease: Bridging the Translational Gap Through Systems-Level Integration

**DOI:** 10.3390/biomedicines14071609

**Published:** 2026-07-17

**Authors:** Che Mohd Nasril Che Mohd Nassir, Thirupathirao Vishnumukkala, Prarthana Kalerammana Gopalakrishna, Saravanan Jagadeesan, Nurul Huda Mohd Nor, Muhammad Zulfadli Mehat, Mohamad Aris Mohd Moklas, Zaw Myo Hein, Mohd Amir Kamaruzzaman

**Affiliations:** 1 Department of Anatomy and Physiology, Faculty of Medicine, Universiti Sultan Zainal Abidin, Kuala Terengganu 20400, Terengganu, Malaysia; nasrilnassir@unisza.edu.my; 2 Department of Human Anatomy, Faculty of Medicine and Health Sciences, Universiti Putra Malaysia, Serdang 43400, Selangor, Malaysia; thirupathirao@imu.edu.my (T.V.); hudamohdnor@upm.edu.my (N.H.M.N.); m_zulfadli@upm.edu.my (M.Z.M.); aris@upm.edu.my (M.A.M.M.); 3 Anatomy Discipline, Human Biology Division, School of Medicine, IMU University, Kuala Lumpur 57000, Malaysia; 4 Physiology Discipline, Human Biology Division, School of Medicine, IMU University, Kuala Lumpur 57000, Malaysia; prarthana@imu.edu.my; 5 Department of Anatomy, School of Medicine, Taylor’s University, Lakeside Campus, Subang Jaya 47500, Selangor, Malaysia; mljsaravanan@gmail.com; 6 Department of Basic Medical Sciences, College of Medicine, Ajman University, Ajman P.O. Box 346, United Arab Emirates; z.hein@ajman.ac.ae; 7 Department of Anatomy, Faculty of Medicine, Universiti Malaya, Kuala Lumpur 50603, Malaysia; 8 Department of Anatomy, Faculty of Medicine, Universiti Kebangsaan Malaysia (UKM), Jalan Yaacob Latif, Bandar Tun Razak, Cheras, Kuala Lumpur 56000, Malaysia

**Keywords:** Alzheimer’s disease, animal model, neurodegenerative disorder, drug development, therapy

## Abstract

Alzheimer’s disease (AD) is a multifactorial neurodegenerative disorder and a leading cause of dementia worldwide, yet effective disease-modifying therapies remain elusive. Rodent models have been indispensable for elucidating key pathological mechanisms, including amyloid-beta (Aβ) deposition, tau pathology, neuroinflammation, and synaptic dysfunction. However, despite decades of preclinical success, the translation of therapeutic findings from rodent studies to clinical efficacy in humans has been largely unsuccessful, highlighting critical limitations in current modelling approaches. This narrative review provides a comprehensive and critical evaluation of rodent models of AD, encompassing transgenic, chemically induced, metabolic, inflammatory, and lesion-based paradigms. Rather than presenting these models in isolation, we propose a systems-level framework that categorizes them based on their ability to recapitulate distinct domains of AD pathology, including genetic, environmental, and systemic contributors. By synthesising existing research, highlighting critical gaps, and proposing a tiered minimum-criteria framework for the development of next-generation models, we offer a definitive operational roadmap instead of merely a list of deficiencies. We highlight that most existing models predominantly reflect familial and reductionist aspects of the disease, while failing to capture the complexity of sporadic AD, aging processes, vascular dysfunction, and whole-body interactions. Importantly, we emphasize emerging dimensions that are underrepresented in current rodent models, including glymphatic dysfunction, cerebral small vessel disease, and the microbiota–gut–brain axis, all of which play crucial roles in AD pathogenesis. We further discuss how integrating these factors into next-generation models may improve translational relevance and therapeutic predictability. By synthesizing current evidence and identifying key gaps, we provide a strategic roadmap for the development of more physiologically relevant and translationally robust rodent models. Advancing toward integrative, systems-based approaches will be essential for bridging the persistent gap between preclinical discoveries and clinical success in AD.

## 1. Introduction

Alzheimer’s disease (AD) is a progressive and multifactorial neurodegenerative disorder and the leading cause of dementia worldwide, posing a major and growing public health challenge [1]. Clinically, AD is characterized by a gradual decline in memory, cognitive function, and executive abilities, often accompanied by behavioural and psychological disturbances that significantly impair daily functioning and quality of life [2,3]. First described in 1906 by Alois Alzheimer following the observation of profound cognitive and personality changes in a middle-aged patient, the disease was later neuropathologically defined by the presence of extracellular amyloid-beta (Aβ) plaques and intracellular neurofibrillary tangles (NFTs), which remain its classical hallmarks [4].

AD can be broadly classified into early-onset familial AD and late-onset sporadic AD. Familial AD, which accounts for less than 5% of cases, is driven by mutations in genes such as amyloid precursor protein (APP) and presenilins (*PSEN1*/*PSEN2*), leading to increased production and aggregation of Aβ peptides [5]. In contrast, sporadic AD represents the vast majority of cases and is influenced by a complex interplay of genetic susceptibility, aging, environmental exposures (exposomes), and systemic factors, with apolipoprotein E (APOE) ε4 being the strongest known genetic risk factor [6]. Despite extensive research, the precise mechanisms underlying sporadic AD remain incompletely understood, reflecting the heterogeneous and multifactorial nature of the disease.

The global burden of AD is rapidly escalating, with more than 50 million individuals currently affected and projections estimating a rise to over 150 million by 2050 [7]. Beyond its clinical impact, AD imposes a substantial socioeconomic burden, with healthcare costs expected to nearly double in the coming decade, underscoring its recognition as a global health priority by the World Health Organization (WHO) [8,9]. Despite decades of intensive research and therapeutic development, effective disease-modifying treatments remain elusive, with most available interventions providing only symptomatic relief.

Rodent models (rats and mice) have been instrumental in advancing our understanding of AD pathophysiology and in preclinical drug development, enabling the investigation of key mechanisms such as Aβ accumulation, tau pathology, neuroinflammation, and synaptic dysfunction. However, a striking disconnect persists between promising preclinical findings and clinical success, as the majority of therapeutics that demonstrate efficacy in rodent models fail in human trials [10]. This translational gap highlights fundamental limitations in current modelling approaches.

Emerging evidence suggests that these limitations extend beyond species differences and instead reflect a broader conceptual challenge: the reliance on reductionist models to study a disease that is inherently complex and systemic [11]. AD is increasingly recognized not only as a disorder of protein aggregation but also as a condition involving vascular dysfunction, metabolic alterations, immune dysregulation, and impaired brain–body interactions [12]. Therefore, there is a critical need to re-evaluate existing rodent models within a systems-level framework that better captures the disease’s multifactorial nature.

In this context, the present review provides a comprehensive and critical reappraisal of rodent models used in AD research. By integrating genetic, induced, and systemic modelling approaches and highlighting emerging dimensions such as glymphatic dysfunction, cerebral small-vessel disease, and the microbiota–gut–brain axis, this review aims to identify key gaps and propose strategies to enhance the translational relevance of preclinical models. Unlike previous reviews that primarily catalogue available AD rodent models, this review adopts a systems-level perspective to evaluate their translational relevance, integrates emerging disease dimensions such as glymphatic dysfunction, cerebral small vessel disease, and the microbiota–gut–brain axis, and proposes a framework for the development of next-generation multifactorial models. Ultimately, a shift toward integrative, systems-based modelling may be essential for bridging the persistent gap between experimental discoveries and clinical success in AD.

### Review Methodology and Scope

This narrative review was performed utilising a structured literature search across PubMed/MEDLINE, Scopus, EBSCOhost, and Web of Science. Search terms encompassed MeSH headings (‘Alzheimer disease/animal models’, ‘transgenic mice/Alzheimer’, ‘streptozotocin/intracerebroventricular’, ‘glymphatic system’, ‘gut-brain axis/neurodegeneration’, ‘cerebral small vessel disease/dementia’) in conjunction with free-text terms (‘rodent model’, ‘translational gap’, ‘sporadic AD’, ‘multi-hit model’, ‘APOE4’, ‘aquaporin-4’). The core search encompassed the period from inception to March 2026, supplemented by specific searches for earlier fundamental works as necessary. The inclusion criteria consisted of original research articles and peer-reviewed reviews in English that reported behavioural and/or neuropathological outcome data in mouse models of AD. Conference abstracts and non-peer-reviewed preprints were omitted. The synthesis is narrative instead of methodical, and we recognise the associated risk of confirmation bias. In areas of mechanistic uncertainty, especially regarding emerging mechanisms like glymphatic dysfunction and the microbiota–gut–brain axis, we have utilised cautious language (‘emerging evidence suggests’, ‘may contribute’) and have explicitly addressed contradictory findings in the pertinent subsections (Section 4.1 and Section 4.3). The application of artificial intelligence (AI) for language editing is mentioned in the Acknowledgements.

## 2. Pathophysiology of AD: From Hypotheses to Systems Biology

Extensive research has been conducted to elucidate the pathogenesis of AD, particularly focusing on its hallmark pathological features, Aβ plaques, and NFTs. Despite decades of investigation, the precise mechanisms underlying disease initiation and progression remain incompletely understood [4]. Experimental studies, particularly in animal models, have generated several classical pathogenesis hypotheses [13]. Recent integrative analyses further emphasize that AD is a multifactorial disorder involving not only protein aggregation but also metabolic dysregulation, vascular impairment, immune activation, and systemic alterations that collectively shape disease trajectory [13].

**Figure 1:**
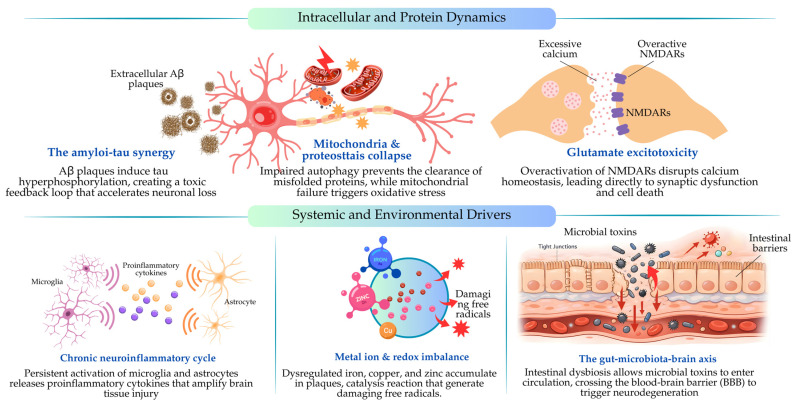
Systems biology overview of Alzheimer’s disease (AD) pathogenesis. The schematic illustrates the interconnected intracellular, systemic, and environmental drivers contributing to neurodegeneration. Top panel: (i) intracellular and protein dynamics amyloid-tau synergy: Extracellular Aβ plaques trigger tau hyperphosphorylation, establishing a toxic feedback loop that accelerates neuronal loss. (ii) Mitochondria and proteostasis collapse: Impaired autophagy prevents the clearance of misfolded proteins, while mitochondrial dysfunction increases oxidative stress. (iii) Glutamate excitotoxicity: Overactivation of N-methyl-D-aspartate receptors (NMDARs) disrupts calcium (Ca^2+^) homeostasis, leading to synaptic failure and apoptosis. Bottom panel: Systemic and environmental drivers. (i) Chronic neuroinflammatory cycle: Persistent activation of microglia and astrocytes releases proinflammatory cytokines, amplifying tissue injury. (ii) Metal ion and redox imbalance: Accumulation of dysregulated iron (Fe), copper (Cu), and zinc (Zn) catalyses the generation of damaging free radicals. (iii) Gut–microbiota–brain axis: Intestinal dysbiosis allows microbial toxins to breach the intestinal barrier and the blood–brain barrier (BBB), triggering central neuroinflammation.

Traditionally, these hypotheses, including cholinergic dysfunction, amyloid cascade, tau pathology, mitochondrial impairment, neuroinflammation, and others, have been considered as independent mechanisms. However, emerging evidence suggests that AD is not driven by a single pathological pathway but rather represents a complex, interconnected network of molecular, cellular, and systemic processes (Figure 1). Contemporary studies highlight that these pathways are tightly interwoven through feedback loops involving oxidative stress, impaired proteostasis, and chronic inflammation, ultimately converging to promote neuronal dysfunction, synaptic loss, and progressive neurodegeneration. Therefore, a systems-level perspective is essential to understand how these mechanisms interact and collectively contribute to disease progression.

### 2.1. Neurotransmitter Dysfunction: Cholinergic and Glutamatergic Pathways

One of the earliest proposed mechanisms of AD was the cholinergic hypothesis, which attributes cognitive decline to reduced cholinergic neurotransmission in the brain. Experimental studies demonstrated decreased acetylcholine levels and degeneration of basal forebrain cholinergic neurons [14], leading to the development of acetylcholinesterase inhibitors such as donepezil, which provide symptomatic relief but do not alter disease progression [15,16]. Critically for rodent modelling, cholinergic deficit is directly relevant to the cholinergic lesion and scopolamine model paradigms reviewed in Section 3.2 [14].

Similarly, the glutamate excitotoxicity hypothesis highlights the role of excessive activation of N-methyl-D-aspartate receptors (NMDARs), resulting in dysregulated calcium (Ca^2+^) homeostasis, synaptic dysfunction, and neuronal death [17,18]. Chronic excitotoxic signalling contributes to mitochondrial overload, oxidative stress, and activation of apoptotic cascades. This led to the development of memantine, an NMDAR antagonist, which also offers limited symptomatic benefit [17]. These neurotransmitter-centric models are mechanistically significant yet only encompass initial synaptic dysfunction, neglecting upstream multifactorial determinants a critical weakness examined in Section 3.

### 2.2. Protein Aggregation Pathways: Amyloid and Tau Hypotheses

The amyloid cascade hypothesis has long dominated AD research, proposing that abnormal cleavage of APP by β- and γ-secretases generates toxic Aβ peptides (particularly Aβ40–42), which aggregate into oligomers and fibrils, forming extracellular plaques that disrupt synaptic function and trigger downstream neurodegenerative processes [19,20]. Importantly, soluble Aβ oligomers are now considered more neurotoxic than insoluble plaques, as they directly impair synaptic signalling and plasticity. Complementing this, the tau hypothesis focuses on the hyperphosphorylation of tau, a microtubule-associated protein essential for axonal transport. In AD, dysregulated kinase activity (e.g., GSK-3β, CDK5) and impaired phosphatase function lead to tau detachment from microtubules, aggregation into NFTs, and disruption of neuronal integrity [21].

Tau pathology is strongly correlated with disease severity and cognitive decline. Importantly, these two pathways are increasingly recognized as interconnected rather than independent. Aβ accumulation can induce tau hyperphosphorylation and aggregation, while pathological tau can exacerbate Aβ toxicity, forming a synergistic cascade that accelerates synaptic dysfunction and neuronal loss.

### 2.3. Mitochondrial Dysfunction and Oxidative Stress, Neuroinflammation, and Systemic Contributors

Numerous supplementary pathogenic pathways merge in Alzheimer’s disease. Mitochondrial dysfunction produces excessive reactive oxygen species (ROS) that harm neurones and facilitate Aβ aggregation and tau hyperphosphorylation through feedback mechanisms [22,23]. When antioxidant defences (GPx, SOD, CAT) are surpassed, calcium homeostasis is compromised, leading to apoptotic activation [24]. Neuroinflammation, induced by persistent activation of microglia and astrocytes, produces proinflammatory cytokines (TNF-α, IL-1β, IL-6) and activates NLRP3 inflammasome pathways, hence exacerbating protein aggregation [25,26,27]. Dysregulation of metal ions (Fe^2+^, Cu^2+^, Zn^2+^) catalyses the production of reactive oxygen species (ROS) and directly affects the kinetics of Aβ aggregation [28,29,30]. The microbiota–gut–brain axis influences systemic inflammation through dysbiosis: the compromise of intestinal barrier integrity permits microbial products (e.g., lipopolysaccharides) to enter the bloodstream, traverse the blood–brain barrier (BBB), and initiate neuroinflammatory responses [31,32,33]. Ultimately, compromised proteostasis involving dysfunctional autophagy, ubiquitin-proteasome system (UPS) impairment, and lysosomal failure obstructs the elimination of Aβ and hyperphosphorylated tau, hence exacerbating neurodegenerative mechanisms [34,35]. These mechanisms collectively demonstrate that AD results from the convergence of interrelated pathways that create a self-reinforcing network. This has significant implications for experimental modelling: most rodent models replicate isolated components of this network rather than the comprehensive system, a fundamental limitation discussed throughout this review.

## 3. Rodent Models in AD: Strengths, Limitations, and Translational Challenges

### 3.1. Role of Rodents in AD Research

Rodents, particularly mice and rats, have become the cornerstone of modern biomedical research due to their genetic tractability, physiological similarities to humans, and experimental versatility. Their widespread adoption has contributed significantly to major scientific advances, including the development of vaccines, elucidation of disease mechanisms, and preclinical drug discovery. Indeed, the availability of fully sequenced genomes, with approximately 95% homology in protein-coding genes between rodents and humans, has enabled the modelling of numerous human diseases at the molecular level [36,37].

However, despite their central role, the reliance on rodent models in biomedical research is not without substantial limitations. While genetic similarity is often cited as a primary justification, it does not necessarily translate into functional equivalence. Differences in gene regulation, epigenetic modifications, immune responses, and metabolic pathways can result in significant divergence in disease manifestation and therapeutic response between rodents and humans. This discrepancy is particularly relevant in complex, multifactorial diseases such as AD, where subtle systemic interactions play a critical role.

Furthermore, the controlled laboratory environment in which rodents are maintained, characterized by uniform diet, minimal environmental stressors, and genetic homogeneity, fails to replicate the heterogeneity of human populations [38]. Factors such as aging, comorbidities, lifestyle, and environmental exposures, which are central to disease development in humans, are often absent or artificially induced in rodent studies. Consequently, rodent models may oversimplify disease processes, leading to an incomplete or biased understanding of pathophysiology.

Another critical consideration is the issue of translational validity. While rodent studies frequently demonstrate promising therapeutic outcomes, these findings often fail to replicate in human clinical trials [39]. This translational gap raises important questions about the predictive reliability of rodent models. In many cases, interventions that target specific molecular pathways show efficacy in rodents but do not produce meaningful clinical benefits in humans, suggesting that these models may not adequately capture the complexity of human disease [39].

Despite these challenges, rodents remain indispensable in biomedical research due to their practical advantages, including short lifespans, rapid breeding cycles, and cost-effectiveness. Their use is particularly valuable for mechanistic studies, genetic manipulation, and early-stage therapeutic screening. However, their limitations must be critically acknowledged, and findings derived from rodent models should be interpreted with caution, particularly when extrapolating to human disease. Thus, rather than viewing rodents as definitive models of human pathology, it is more appropriate to consider them as experimental systems that capture selected aspects of disease biology. The challenge moving forward lies in refining these models to better reflect the complexity, heterogeneity, and systemic nature of human diseases.

**Figure 2:**
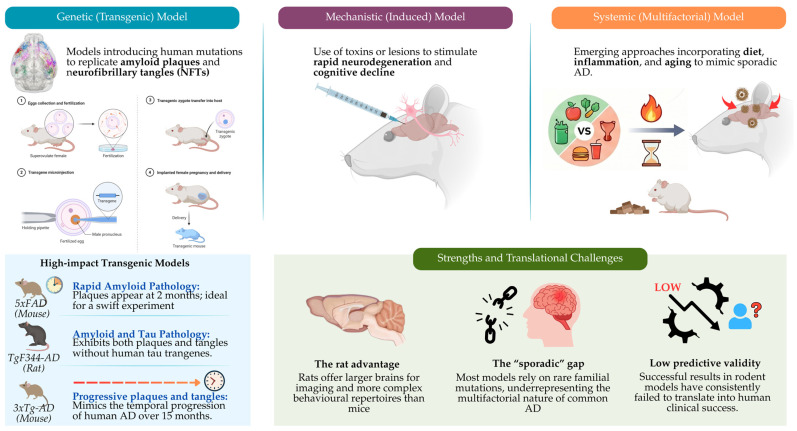
Overview of rodent models in Alzheimer’s disease (AD) Research. This schematic categorizes the primary experimental approaches, genetic, mechanistic, and systemic, used to simulate AD pathology in rodents. Genetic models utilize human mutations to replicate hallmark amyloid and tau pathologies, with specific high-impact examples like the 5xFAD mouse for rapid onset and the 3xTg-AD mouse for temporal progression. Mechanistic models employ toxins or lesions to induce acute neurodegeneration, while systemic models integrate lifestyle factors like diet and aging to better mimic sporadic AD. The figure also highlights critical translational considerations, noting the anatomical advantages of rat models alongside the “sporadic gap” and the historically low predictive validity of these models in human clinical trials.

Despite the diversity of available models and decades of research, a fundamental limitation persists: no single rodent model is capable of fully recapitulating the complexity of human AD. This limitation is particularly evident in the context of sporadic AD, which accounts for the vast majority of cases and arises from a multifactorial interplay of genetic, environmental, vascular, and metabolic factors. To critically evaluate these models, it is useful to categorize them based on their underlying design and the aspects of disease they aim to replicate. Broadly, rodent models can be classified into genetic (transgenic), induced (mechanistic), and systemic (multifactorial) models (Figure 2). The relative merit of each model class is context-dependent, not absolute. Transgenic models exhibit excellent construct validity for familial AD pathways but low prediction validity for sporadic AD and non-amyloid treatment targets. Toxin-based models serve as efficient and economical screening instruments for mechanistic hypotheses, although they exhibit limited face validity for the complete AD phenotype. This distinction that model utility is contingent upon the specific question rather than being inherently superior or inferior is upheld throughout the subsequent review.

### 3.2. Genetic (Transgenic) Models: Overrepresentation of Amyloid-Centric Pathology

#### 3.2.1. Transgenic Rat Models

Transgenic rat models are invaluable tools in AD research, providing insights into the disease’s molecular mechanisms and potential treatments. They have genetic and physiological similarities to humans and more complex behaviours compared to mice, making rats ideal AD models [40]. Early models like UKUR25 and McGill-R-Thy1-APP expressed human amyloid precursor protein (hAPP) with familial AD mutations but only showed intracellular Aβ accumulation, not amyloid plaques [41,42]. The Tg478/Tg1116 model was the first to develop amyloid plaques, albeit late in life [43]. The enhanced PSAPP model introduced a presenilin mutation, leading to earlier plaque formation and issues like hypertension and kidney disease [44]. The McGill-R-Thy1-APP rat, which mimics amyloid pathology in AD-relevant brain regions, and the bi-genic TgF344-AD rat, which shows both amyloid plaques and NFT-like structures, are standout models. The TgF344-AD rat, notable for replicating plaque and tangle pathology without a human tau transgene, may achieve this through rat tau splicing into human-equivalent isoforms [43].

Newer models that express human tau with frontotemporal dementia mutations now exhibit tau tangles and cognitive deficits, further advancing AD research [45]. These transgenic rat models, with their larger brains and more complex behaviours, offer a unique opportunity to investigate AD’s intricate pathology, contributing significantly to the understanding of both amyloid and tau-related mechanisms and paving the way for the development of potential treatments. Transgenic rat models represent a complementary and increasingly important extension of traditional mouse-based systems, offering distinct advantages in modelling AD pathology. While transgenic mice such as APP/PS1, 5xFAD, and 3xTg-AD have historically dominated the field [46], rat models provide enhanced neuroanatomical complexity, improved behavioural resolution, and greater translational relevance in certain experimental contexts. These models have been developed through the introduction of human genes associated with amyloid and tau pathology, enabling the study of key molecular processes underlying AD.

One of the primary strengths of transgenic rat models lies in their ability to more closely replicate aspects of human brain organization and cognitive function. Their larger brain size facilitates advanced imaging, electrophysiological recordings, and surgical manipulations, while their more sophisticated behavioural repertoire allows for nuanced assessment of learning, memory, and executive function [47]. Models such as the TgF344-AD rat, which exhibits both amyloid plaques and tau-like pathology, represent a significant advancement by capturing multiple hallmarks of AD within a single system [43]. Despite these advantages, transgenic rat models share many of the fundamental limitations observed in mouse models. Most notably, they rely on genetic mutations associated with familial AD, which accounts for only a small fraction of human cases. Consequently, these models tend to overemphasize amyloid-driven mechanisms while underrepresenting the multifactorial nature of sporadic AD, including aging, vascular contributions, metabolic dysfunction, and environmental influences.

Additionally, the overexpression of mutant proteins often leads to accelerated and non-physiological disease progression, which may not accurately reflect the gradual onset and complexity of AD in humans [48]. While some rat models demonstrate improved representation of tau pathology and neurodegeneration, inconsistencies remain in fully reproducing the spectrum of human disease features [49]. Furthermore, as with mouse models, therapeutic strategies that show promise in transgenic rats have not consistently translated into clinical success, highlighting persistent concerns regarding predictive validity [39]. In summary, transgenic rat models provide valuable opportunities to investigate AD pathology with enhanced behavioural and anatomical fidelity. However, their reliance on familial mutations and reductionist design limits their ability to fully capture the complexity of sporadic AD. As such, they should be viewed as complementary tools within a broader experimental framework, rather than definitive models of human disease.

#### 3.2.2. Transgenic Mouse Models

**Figure 3:**
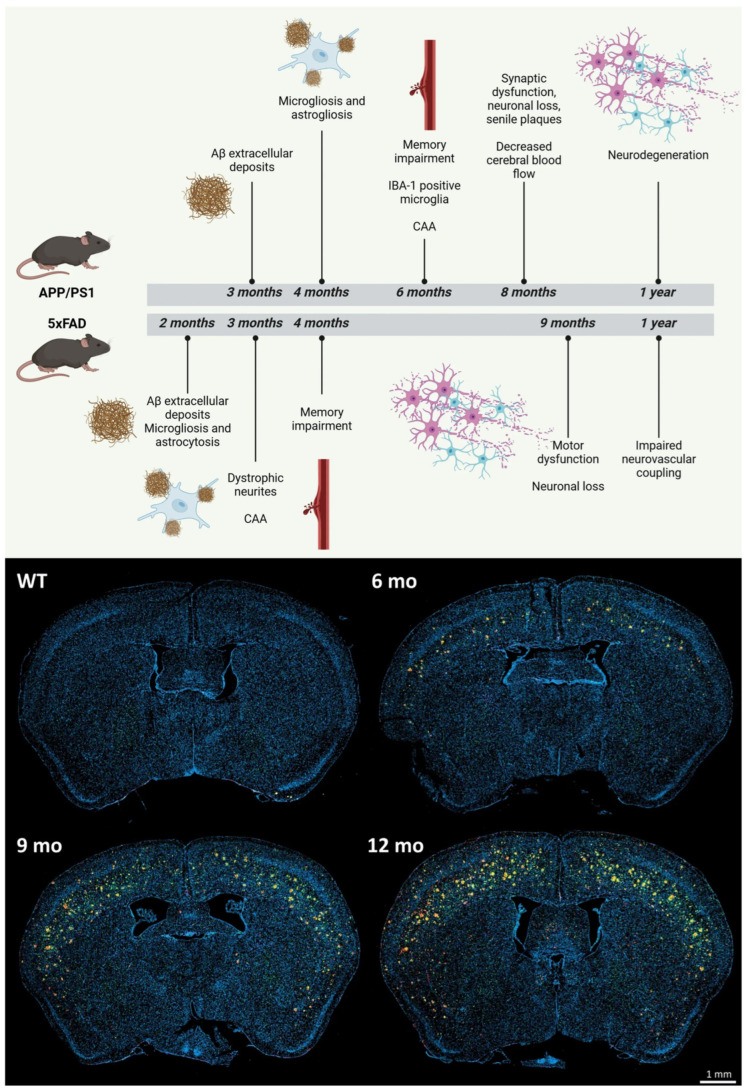
Comparative timeline and neuropathological progression of APP/PS1 and 5xFAD mouse models of Alzheimer’s disease (AD). The top panel illustrates the temporal onset of key pathological features, showing earlier and more aggressive amyloid-β (Aβ) deposition, gliosis, and cognitive impairment in 5xFAD mice compared to APP/PS1 mice, which exhibit a more gradual progression, including cerebrovascular alterations and reduced cerebral blood flow. The bottom panel presents representative coronal brain sections from APP/PS1 mice and wild-type controls at different ages, highlighting progressive Aβ plaque accumulation (red) alongside microglial activation (Iba-1, green), astrocytosis (GFAP, violet), and nuclear staining (DAPI, blue) over time. Images adapted from Pérez-Medina-Carballo et al. [61].

Numerous transgenic mouse models have emerged as essential instruments in AD research, each reflecting distinct facets of AD’s pathology. The APP/PS1 double transgenic mouse model simultaneously expresses the Swedish mutant of human amyloid precursor protein (APPswe) and the ΔE9 variant of human presenilin 1 (PS1-ΔE9) [50]. This model manifests amyloid plaques as early as 6 months, demonstrates cognitive impairments, and exhibits neuroinflammation, rendering it one of the most prevalent models for evaluating anti-amyloid treatments [51,52]. The expedited pathology facilitates comparatively swift experimental durations (Figure 3).

The 5xFAD model contains five familial AD mutations: APP K670N/M671L (Swedish), APP I716V (Florida), APP V717I (London), PSEN1 M146L, and PSEN1 L286V [53]. This animal exhibits rapid and pronounced amyloid pathology, with plaques detectable at 2 months of age, accompanied by synaptic dysfunction, neuronal death, and behavioural impairments by 4–6 months. The swift advancement renders it especially advantageous for therapeutic intervention research [54,55]. The triple transgenic 3xTg-AD model expresses mutant APP, PS1, and tau (P301L), resulting in the gradual, age-dependent formation of plaques and tangles [56]. Plaques manifest at six months, succeeded by tau pathology at twelve to fifteen months, closely resembling the temporal pattern observed in human AD. This animal demonstrates synaptic degeneration, cognitive impairments, and behavioural anomalies, rendering it suitable for exploring the interaction between amyloid and tau diseases [57].

Finally, the P301S mice, which express the human tau mutation linked to frontotemporal dementia, serve as pure tauopathy models, exhibiting significant NFTs, neurodegeneration, and impairments in motor and cognitive functions [58]. Additionally, the rTg4510 model exhibits adjustable tau expression, enabling temporal regulation of tau disease and indicating that tau-mediated neurodegeneration can be reversed if tau expression is sufficiently repressed at an early stage [59].

#### 3.2.3. Sporadic AD Modelling: APOE4 Knock-In, Humanized Models, and the Limits of Overexpression Systems

Most of the transgenic rodent models mentioned previously were developed from mutations associated with familial Alzheimer’s disease (FAD), accounting for less than 5% of all AD cases. This significant discrepancy is particularly relevant, as the pathophysiological mechanisms underlying sporadic, late-onset Alzheimer’s disease which impacts over 95% of individuals differ from the amyloid-overproduction model represented by APP/PS1, 5xFAD, or 3xTg-AD mice. Intermittent AD is influenced by the interplay of ageing, APOE genotype, vascular and metabolic risk factors, and environmental exposures. Four under-represented modelling techniques warrant concentrated study [8].

Models associated with APOE4: APOE4 allele constitutes the most significant genetic risk factor for sporadic AD, elevating lifetime risk around threefold in heterozygous carriers and eight- to twelvefold in homozygous carriers relative to APOE3 [9]. APOE4 diminishes Aβ clearance, amplifies tau pathology, triggers blood–brain barrier disruption, and intensifies neuroinflammation via distinct cellular mechanisms [10]. Nonetheless, APOE4 is absent in the majority of traditional transgenic models. Knock-in (KI) models, including the APOE4-KI and APOE-TR (targeted-replacement) lines, in which human APOE4 substitutes murine Apoe under the endogenous promoter, eliminate the confounding factors of transgenic overexpression and allow for physiologically regulated expression [11]. Models crossed onto amyloidogenic backgrounds (e.g., 5xFAD × APOE4-KI) demonstrate increased amyloid deposition, diminished Aβ clearance, and heightened neuroinflammatory responses relative to their APOE3 equivalents, thereby more accurately reflecting the risk framework of human sporadic AD [12]. Significantly, APOE4 interacts with biological sex: female APOE4 carriers face a disproportionately higher risk than their male counterparts, a relationship that has only begun to be systematically incorporated into rodent studies [13]. According to Section 6.1, APOE4-KI must be incorporated as a Tier 3 minimum criterion in forthcoming models designed to guide sporadic AD therapies.

Age constitutes the primary risk factor. Ageing, the paramount risk factor for sporadic AD, is frequently overlooked in preclinical models. Transgenic models typically exhibit AD-like pathology between 3 and 9 months of age, resulting from an artificial genetic excess rather than the gradual accumulation of risk over decades. This distinction is crucial: late-onset AD in the human brain transpires against a backdrop of age-associated declines in proteostasis, mitochondrial function, glymphatic clearance efficiency, immunological surveillance, and cerebrovascular integrity, none of which are replicated in young transgenic mice [14]. In the Tier 2 criteria of our framework (Section 6.2), we advocate for the utilisation of naturally aged rodents (≥18 months for mice; ≥24 months for rats), which exhibit nuanced cognitive deficits and neurobiological alterations that more accurately reflect the initial phases of sporadic AD, thereby offering a more physiologically relevant foundation for evaluating interventions targeting authentic disease modification.

Models of vascular and blood–brain barrier dysfunction: The neurovascular unit, consisting of cerebral endothelium, pericytes, astrocytic end-feet, and the associated basement membrane, is increasingly acknowledged as a principal source of dysfunction in sporadic AD. BBB disruption precedes significant amyloid deposition in certain sporadic AD patients, while sustained cerebral hypoperfusion contributes to tau pathology and impedes glymphatic waste clearance, regardless of amyloid presence. Specialised vascular models, such as the bilateral carotid artery stenosis (BCAS) model of chronic hypoperfusion, angiotensin II models of hypertension, and pericyte-deficient PDGFR-β heterozygous mice, replicate the vascular characteristics of neurodegeneration, leading to white matter lesions, BBB compromise, and cognitive deficits [11]. These models provide a framework for examining the connections between CSVD and AD (Section 4.2). Their incorporation with amyloidogenic or tau backgrounds would produce more translatable multi-hit models that could replicate the vascular–neurodegeneration co-pathology characteristic of the majority of human AD patients at autopsy.

Humanised knock-in and overexpression systems: There is growing evidence of the artificial consequences of transgenic overexpression. The overexpression of APP produces excessive amounts of C-terminal fragments (CTFs), soluble APPα/β, and intracellular APP domains fragments that exhibit distinct neurotoxic and synaptic effects which are either absent or present in minimal levels in human AD. The App knock-in (AppNL-G-F) model developed by Cavedo et al. (2017) overcomes this limitation by incorporating FAD mutations into the endogenous App locus under physiological promoter regulation, producing Aβ pathology without APP overexpression [15]. These models suggest that overexpression artefacts may constitute a significant portion of the synaptic and inflammatory phenotypes observed in typical transgenic mice, rather than accurately representing authentic AD biology. AppNL-G-F mice, incorporating FAD mutations, enhance methodological rigour by improving construct validity and diminishing non-specific transcriptome noise, a concern increasingly highlighted by single-cell RNA sequencing analyses across several model systems [15].

### 3.3. Mechanistic (Lesion/Toxin)-Induced Models

Lesion-induced and toxin-induced models employ chemical agents and environmental toxins to selectively damage brain regions critical for learning and memory, particularly the hippocampus, thereby inducing AD-like neurodegeneration. These models are designed to mimic specific pathological features of AD, including cognitive deficits, neuroinflammation, neuronal loss, and biochemical alterations [60]. They are particularly useful for studying mechanisms such as cholinergic dysfunction, oxidative stress, and neurotoxicity, although they often lack the progressive and multifactorial nature of human AD.

Moreover, neurotoxin-induced models are widely used to investigate specific mechanisms of neurodegeneration relevant to AD. Agents such as okadaic acid, colchicine, trimethyltin (TMT), kainic acid, and ibotenic acid induce neuronal loss, oxidative stress, neuroinflammation, synaptic dysfunction, and cognitive impairment. For example, okadaic acid inhibits protein phosphatases PP1 and PP2A, resulting in tau hyperphosphorylation and neurofibrillary tangle-like pathology, whereas colchicine disrupts microtubule assembly and axonal transport, leading to hippocampal degeneration and memory deficits. Although these models are useful for studying discrete pathogenic mechanisms and evaluating neuroprotective interventions, they generally lack the progressive, multifactorial, and age-dependent features characteristic of human AD. Consequently, neurotoxin-induced models are best viewed as mechanistic tools rather than comprehensive representations of the disease.

#### 3.3.1. Intracerebroventricular (ICV) Streptozotocin (STZ) Model

The intracerebroventricular (ICV) streptozotocin (STZ) model is widely employed to investigate sporadic AD-like pathology, particularly in relation to brain insulin resistance and metabolic dysfunction [62]. STZ, an alkylating agent derived from *Streptomyces achromogenes*, is administered directly into the cerebral ventricles, enabling localized neurotoxicity while minimizing systemic exposure. This approach induces key AD-like features, including cognitive impairment, neuroinflammation, oxidative stress, and alterations in amyloid metabolism [62].

Importantly, the pathological profile of this model is highly dose-dependent, with distinct phenotypic outcomes observed across dosing regimens (Table 1). Lower doses (≤1 mg/kg) induce subtle metabolic and cognitive alterations, making them suitable for modelling early-stage or prodromal AD [63,64]. In contrast, intermediate doses (~2 mg/kg), typically administered in split injections, produce more robust cognitive deficits, neuroinflammation, and cholinergic dysfunction and are therefore most used to approximate sporadic AD-like pathology [65]. Higher doses (≥3 mg/kg), however, result in acute neurotoxicity and widespread neuronal damage, which more closely resemble non-specific brain injury rather than progressive neurodegeneration [63,66].

Despite its utility, the ICV-STZ model presents several limitations. The rapid induction of pathology contrasts with the slow progression of human AD, and the underlying mechanisms, primarily driven by insulin signalling impairment, capture only a subset of disease processes. Furthermore, key features such as robust tau pathology and complex vascular contributions are not consistently reproduced. Nevertheless, at intermediate doses (1–2 mg/kg), the ICV-STZ model currently represents the most practical single-model approximation of sporadic AD metabolic pathology, offering the best available approximation of brain insulin resistance as a disease axis. Its greatest strength lies not in replicating the full spectrum of AD pathology but in modelling a specific disease axis, which should be interpreted within a broader, multi-hit framework.

#### 3.3.2. Aβ Injection Model

The Aβ injection model is a well-established method for inducing AD-like pathology in animal studies. This approach involves the direct administration of aggregated Aβ peptides into specific brain regions, such as the hippocampus or ventricles, to simulate amyloid plaque formation and associated neurotoxicity. Commonly used Aβ species include Aβ40, Aβ42, and Aβ25–35, which are introduced via intrahippocampal, intracerebral, or ICV injections [68]. This model enables rapid induction of pathological and behavioural changes, including cholinergic dysfunction and memory impairment, allowing for shorter experimental timelines compared to transgenic models [69]. It is particularly useful for studying the direct effects of Aβ toxicity and for evaluating therapeutic agents targeting amyloid-related pathways.

However, the Aβ injection model has significant limitations. It typically requires supraphysiological concentrations of Aβ, which may not accurately reflect endogenous levels observed in AD patients [70]. Additionally, this model does not consistently reproduce key pathological features such as NFTs, limiting its ability to represent the full spectrum of AD pathology. The acute nature of Aβ administration also fails to capture the chronic and progressive development of the disease.

#### 3.3.3. Scopolamine-Induced Model

The scopolamine-induced model is a commonly employed pharmacological instrument for simulating AD symptoms, particularly focusing on cognitive deficits in learning and memory [71]. Scopolamine functions as a nonselective antagonist of muscarinic acetylcholine receptors (mAChRs), hence impairing the cholinergic signalling pathway essential for memory formation and retrieval. This disturbance results in cognitive abnormalities that resemble the initial phases of cognitive decline linked to AD [72].

Intraperitoneal administration of scopolamine elicits many pathological alterations in the brain, encompassing increased oxidative stress and cholinergic impairment. This leads to the accumulation of Aβ and inflammatory markers in the hippocampus, a crucial area for memory and learning, hence worsening cognitive deficits [73]. This model is very useful for assessing prospective neuroprotective drugs, as numerous studies have shown that these compounds can improve cognitive performance by augmenting antioxidant defences and maintaining cholinergic activity in scopolamine-treated rats [74]. The scopolamine-induced model offers a framework for comprehending the mechanisms of cognitive loss in AD and enables the investigation of therapeutic approaches to alleviate these deficits.

#### 3.3.4. Aluminium Chloride Model

The aluminium chloride (AlCl_3_) Model is a crucial experimental method for investigating AD, as it replicates essential pathological features associated with the disorder. Aluminium, recognized as a widespread neurotoxic metal, can cross the BBB and accumulate in the brain, largely due to its high affinity for transferrin receptors. This accumulation has been linked to neuronal damage, primarily through aluminium’s interaction with hyperphosphorylated tau proteins, which contribute to the formation of neurofibrillary tangles, one of the defining characteristics of AD [75].

Notably, elevated levels of aluminium have been detected in the cerebrospinal fluid and brain tissue of individuals with AD [76], further establishing a connection between aluminium exposure and the disease’s pathogenesis. Utilizing the AlCl_3_ model, researchers can observe cognitive deficits and neurodegeneration that closely mirror the symptoms observed in AD, facilitating a deeper understanding of the neurotoxic effects of aluminium. Experimental findings indicate that aluminium accelerates tau aggregation, promotes apoptosis, and worsens motor dysfunction in tauopathy models [77]. Furthermore, aluminium exposure has been shown to enhance Aβ aggregation, induce neuroinflammation, increase oxidative damage, and trigger neuronal apoptosis, significantly impairing cognitive functions in animal models [78].

#### 3.3.5. D-Galactose Model

The D-galactose (D-gal) model is a recognized experimental method for investigating aging and age-related neurodegeneration, such as AD. D-gal, a naturally occurring monosaccharide, functions as an energy source and a component of glycosylation processes in mammals. In excessive amounts, D-gal is converted into aldose and hydroperoxide, resulting in increased formation of ROS that intensifies oxidative stress. Oxidative stress accelerates aging and significantly contributes to neurodegeneration [79]. This model entails the administration of continuous D-gal injections in rodents, leading to numerous detrimental effects, such as progressive impairments in learning and memory, mitochondrial dysfunction, heightened oxidative stress, and the activation of glial cells, including microglia and astrocytes, thereby simulating the natural aging process of the brain [80].

However, the D-gal model provides valuable insights into aging mechanisms but fails to accurately replicate critical features of AD, such as NFTs, amyloid plaques, tau phosphorylation, and the accumulation of Aβ pathology. Studies demonstrate that male mice are more susceptible to oxidative damage and ensuing spatial memory deficits than females, suggesting potential gender differences in the reaction to oxidative stress with aging [81,82].

#### 3.3.6. Combined AlCl_3_ and D-Galactose Model

To overcome the limitations of individual models, researchers have increasingly employed a combined AlCl_3_ and D-gal model to better simulate the multifactorial nature of AD. This dual approach integrates the neurotoxic effects of aluminium with the aging-related oxidative stress induced by D-gal, thereby producing a more comprehensive representation of disease pathology [83]. The combination model enhances oxidative stress, neuroinflammation, and neuronal apoptosis more robustly than either agent alone. D-gal-induced aging creates a vulnerable neural environment characterized by mitochondrial dysfunction and increased ROS production, which is further exacerbated by aluminium accumulation [83]. This synergistic interaction accelerates cognitive decline, promotes Aβ aggregation, and facilitates tau hyperphosphorylation, thereby mimicking both aging-related and AD-specific pathological processes.

Experimental studies have demonstrated that rodents subjected to combined AlCl_3_ and D-gal treatment exhibit more pronounced impairments in learning and memory, along with increased markers of oxidative damage, inflammatory cytokines, and neuronal degeneration [84]. Additionally, this model has been shown to induce both amyloidogenic and tau-related changes, addressing key limitations observed in single-agent models [85]. Despite these advantages, the combined model still relies on artificial induction and may not fully capture the complexity of sporadic AD. Nevertheless, it represents a more integrative approach that bridges the gap between aging and neurotoxicity, offering improved relevance for studying disease mechanisms and evaluating potential therapeutic interventions.

Overall, while lesion-induced models such as the STZ, Aβ injection, and toxin-based approaches provide valuable insights into specific mechanisms of neurodegeneration, they should be interpreted as simplified systems that model isolated aspects of AD rather than comprehensive representations of the disease.

### 3.4. Systemic and Multifactorial Models: Emerging but Incomplete Approaches

Recognizing the limitations of reductionist models, there has been increasing interest in developing systemic models that incorporate broader physiological and environmental factors associated with AD. These include metabolic models (e.g., high-fat diet and insulin resistance), inflammation-based models (e.g., LPS-induced neuroinflammation), microbiota-related models, and surgical lesion approaches that target specific factors [86,87,88]. These models are particularly relevant for studying sporadic AD, as they capture key risk factors such as metabolic syndrome, chronic inflammation, and gut dysbiosis. Within this framework, metabolic models have gained significant attention due to the strong epidemiological and mechanistic links between metabolic dysfunction and AD.

An important limitation of many currently available rodent models is the insufficient incorporation of vascular and metabolic risk factors that characterize the majority of sporadic AD cases. Hypertension, diabetes mellitus, obesity, insulin resistance, and cerebral small vessel disease are increasingly recognized as major contributors to cognitive decline and dementia. These factors promote BBB dysfunction, cerebral hypoperfusion, neuroinflammation, oxidative stress, and impaired glymphatic clearance, thereby accelerating amyloid and tau pathology. Future AD models should therefore integrate vascular and metabolic comorbidities to better reflect the multifactorial nature of human disease and improve translational validity.

#### 3.4.1. Metabolic Models

Metabolic models explore the contribution of systemic metabolic disturbances, particularly obesity and type 2 diabetes, to AD pathogenesis. Among these, the high-fat diet (HFD) model is widely used to simulate metabolic syndrome and its neurological consequences. The HFD model serves as a critical experimental platform for examining how metabolic dysfunction accelerates neurodegenerative processes [89]. Studies have demonstrated that prolonged consumption of HFD leads to obesity, insulin resistance, glucose intolerance, and systemic inflammation, all of which are associated with an increased risk of AD [90]. Importantly, these metabolic disturbances are not confined to peripheral tissues but extend to the central nervous system, where they impair hypothalamic function, a key regulator of energy homeostasis and neuroendocrine signalling [91].

In rodent models, HFD has been shown to exacerbate cognitive decline, promote Aβ deposition, and facilitate tau hyperphosphorylation, thereby recapitulating key pathological features of AD [86]. Additionally, these models reveal notable sex-specific differences: females often exhibit greater weight gain and hypothalamic inflammation, whereas males tend to display more pronounced systemic inflammatory responses [92]. Such findings underscore the importance of considering biological sex as a variable in AD research. Despite their relevance, metabolic models also present limitations. The extent to which diet-induced metabolic changes directly translate to human AD pathology remains uncertain, particularly given differences in lifespan, metabolism, and environmental exposures. Furthermore, while these models effectively capture metabolic contributions to neurodegeneration, they often fail to reproduce the full spectrum of AD pathology in isolation.

#### 3.4.2. Inflammation-Based Models

Chronic neuroinflammation is a central feature of AD, and inflammation-based models aim to replicate this aspect of disease progression. Among these, lipopolysaccharide (LPS)-induced neuroinflammation is one of the most widely used approaches. LPS, a component of Gram-negative bacterial cell walls, activates Toll-like receptor 4 (TLR4) on microglia, astrocytes, and neurons, triggering a robust inflammatory response [88]. This activation leads to the release of proinflammatory cytokines such as IL-1β and TNF-α, mimicking inflammatory cascades observed in AD pathology.

In experimental settings, LPS administration induces neuroinflammation, oxidative stress, and cognitive deficits through activation of key signalling pathways, particularly nuclear factor kappa B (NF-κB). This pathway upregulates inflammatory mediators such as inducible nitric oxide synthase (iNOS) and cyclooxygenase-2 (COX-2), thereby amplifying neuronal damage and contributing to amyloid-related pathology [93,94]. The effects of LPS vary depending on the route of administration. Intraperitoneal injection, for example, leads to systemic inflammation that subsequently impacts the brain, resulting in memory impairment, increased Aβ production, and heightened oxidative stress [93]. While LPS models are valuable for dissecting inflammatory mechanisms and testing anti-inflammatory therapies, they primarily represent acute or subacute inflammatory responses rather than the chronic, progressive inflammation characteristic of AD.

#### 3.4.3. Surgical Models

Surgical models provide another dimension to systemic approaches by targeting specific neural circuits implicated in AD. Among these, cholinergic lesion models are particularly significant. These models involve selective damage to basal forebrain cholinergic neurons, especially within the nucleus basalis magnocellularis, which plays a critical role in learning and memory [95]. In rodent studies, such lesions lead to cognitive deficits accompanied by increased Aβ accumulation and tau hyperphosphorylation in the hippocampus and cortex [96]. Cholinergic lesion models are valuable for understanding the role of neurotransmitter systems in AD and for evaluating therapies aimed at restoring cholinergic function. However, similar to other systemic models, they capture only a subset of disease features and do not fully replicate the multifactorial nature of AD.

Moreover, despite their conceptual advantages, systemic models also have notable limitations. They often lack specificity and may not consistently reproduce classical AD hallmarks such as amyloid plaques and NFTs. Additionally, variability in experimental design, including differences in diet composition, dosing regimens, and duration of exposure, can lead to inconsistent and sometimes conflicting results across studies. Another critical limitation is that many of these models still isolate individual systemic factors rather than integrating multiple interacting pathways. Given that sporadic AD arises from the convergence of metabolic, inflammatory, vascular, and genetic influences, single-factor models remain inherently reductionist despite their broader scope.

Nevertheless, systemic and multifactorial models represent an important step toward a more holistic understanding of AD. Future progress will depend on the development of integrative, multi-hit models that better reflect the complexity, heterogeneity, and progressive nature of human disease. Table 2 summarizes key rodent models used in AD research, highlighting their genetic modifications, induced pathological features, and relevance to different aspects of the disease.

### 3.5. Sex as a Biological Variable in AD Rodent Models

AD demonstrates a significant gender bias, with over two-thirds of those affected being women, a discrepancy that cannot be exclusively explained by differences in lifespan. Women with AD have a more aggressive and rapid progression of tau pathology, more hippocampal shrinkage for a similar amyloid-beta burden, and a quicker transition from mild cognitive impairment to dementia compared to men [16]. In contrast, men with AD demonstrate increased Aβ plaque density at similar cognitive levels and heightened neuroinflammatory microglial activation in specific brain regions. These discrepancies are not merely clinical ascertainment errors; they are increasingly validated at the biology level in mouse models, revealing sex-specific pathways that must be incorporated into experimental design [17].

Regarding sex-specific variations in the aetiology of illnesses in rodent models in the 3xTg-AD mouse model, females exhibit earlier and more widespread amyloid pathology than men, accompanied by increased astrocyte activation and accelerated cognitive deterioration in spatial and associative memory tasks. In the 5xFAD model, female mice demonstrate consistently accelerated and more prominent Aβ deposition, as well as increased intraneuronal Aβ accumulation relative to males, especially in cortical layer 5, a phenomenon linked to the modulation of APP processing by sex hormones. However, male 5xFAD specimens exhibit more significant synapse loss and axonal degeneration at later time intervals. In the APP/PS1 paradigm, females exhibit increased microglial activation, distinguished by a unique activation pattern compared to males, as seen by enhanced TREM2 expression, indicating that sex significantly affects the neuroinflammatory milieu and amyloidogenesis. These findings transcend transgenic models: in the ICV-STZ sporadic AD model, female rats exhibit augmented neuroinflammatory responses and heightened cholinergic vulnerability relative to males at equivalent doses, while the D-galactose ageing model demonstrates that male rodents are more prone to oxidative stress-induced spatial memory deficits (see Section 3.3.5). The data collectively suggest that the nature of sex differences depends on the specific pathways implicated, with females showing more vulnerability to amyloid, tau, and neuroinflammatory pathways, whilst males may be more prone to vascular and oxidative stress processes [20].

Hormonal and Molecular Underpinnings: The molecular foundation of sex-specific biology in AD in mouse models is linked to the interplay of sex hormones, APOE4, and neuroinflammatory signalling. Oestrogen demonstrates many neuroprotective benefits, including the overexpression of Aβ clearance enzymes (neprilysin, IDE), improvement of synaptic plasticity, and attenuation of microglial reactivity. The rapid decrease in oestrogen levels following ovariectomy or natural menopause exacerbates AD-like pathology in female transgenic mice, which is substantially alleviated by oestrogen replacement, but within a restricted and context-dependent period. Two male sex hormones, testosterone and its aromatised metabolite oestradiol, affect Aβ metabolism and tau phosphorylation via androgen receptor signalling, and their age-related reduction in male rodents coincides with heightened vulnerability to neurodegeneration. The interplay between APOE4 and sex is notably important: in humanised APOE4 models, females with APOE4 demonstrate significantly increased blood–brain barrier dysfunction and Aβ accumulation compared to their APOE3 female counterparts and APOE4 males, likely due to sex-specific variations in APOE-mediated lipid metabolism and microglial activity [21].

Taken together, each category of rodent models captures distinct aspects of AD pathophysiology but fails to fully replicate the disease in its entirety. Transgenic models emphasize genetic and amyloid-driven mechanisms, induced models focus on isolated pathological processes, and systemic models incorporate broader physiological interactions. This fragmentation underscores a central challenge in AD research: the reliance on reductionist models to study a fundamentally complex disease. The persistent failure of therapeutic translation from preclinical studies to clinical success may, in part, reflect this mismatch. Future research should therefore prioritize the development of integrative, multi-hit models that combine genetic susceptibility, aging, metabolic dysfunction, and environmental factors. Such approaches may better reflect the heterogeneity of sporadic AD and improve the predictive value of preclinical findings.

## 4. Missing Dimensions in Current Rodent Models of AD

**Figure 4:**
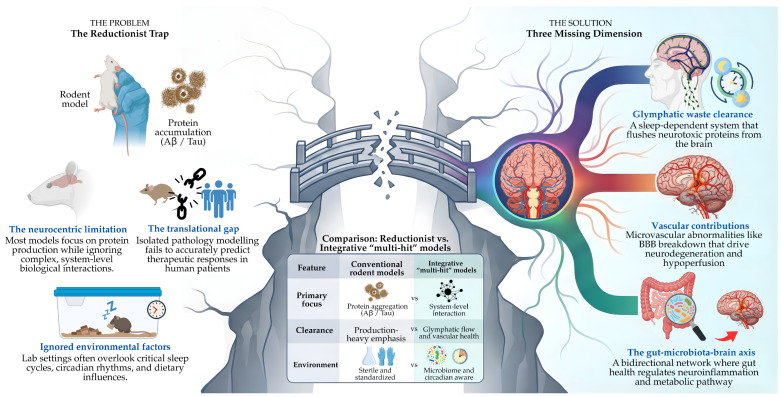
Bridging the gap in Alzheimer’s disease (AD) research. This schematic illustrates the “Reductionist Trap” of conventional rodent models, which primarily focus on neurocentric protein accumulation, e.g., amyloid beta (Aβ) and tau, while ignoring systemic biological interactions. The central comparison table contrasts these traditional approaches with integrative “Multi-Hit” models, which address three critical missing dimensions: glymphatic waste clearance during sleep, vascular contributions such as blood–brain barrier breakdown, and the bidirectional influence of the microbiota–gut–brain axis. By incorporating these systemic and environmental factors, researchers aim to overcome the translational gap between laboratory findings and successful therapeutic outcomes in human clinical trials.

Despite the extensive use of rodent models to investigate AD, most experimental approaches remain reductionist, focusing on isolated pathological features such as Aβ accumulation or tau pathology. However, emerging evidence indicates that AD is a systems-level disorder involving complex interactions between neuronal, vascular, metabolic, and peripheral systems. A critical limitation of current rodent models is their failure to incorporate several key biological dimensions that are increasingly recognized as central to AD pathogenesis. Among these, glymphatic dysfunction, cerebral small vessel disease (CSVD), and the microbiota–gut–brain axis represent underexplored yet highly influential pathways (Figure 4). The omission of these dimensions may contribute significantly to the translational gap between preclinical findings and clinical outcomes.

### 4.1. Glymphatic Dysfunction: The Overlooked Clearance System

The glymphatic system has emerged as a crucial mechanism for the clearance of metabolic waste products, including Aβ and tau, from the brain. This perivascular network facilitates the exchange of cerebrospinal fluid (CSF) and interstitial fluid (ISF), thereby maintaining brain homeostasis. Importantly, glymphatic function is strongly dependent on sleep, particularly slow-wave sleep, during which clearance efficiency is markedly enhanced [97]. Disruption of glymphatic function has been increasingly implicated in AD pathogenesis, with impaired clearance leading to the accumulation of neurotoxic proteins [97,98]. Aging, sleep fragmentation, vascular dysfunction, and astrocytic alterations, particularly involving aquaporin-4 (AQP4) polarization, have all been shown to compromise glymphatic flow [99].

Despite its importance, glymphatic dysfunction is rarely assessed or explicitly modelled in conventional rodent studies. Most transgenic and induced AD models focus on the production of Aβ rather than its clearance, thereby overlooking a critical component of disease biology. Furthermore, standard laboratory conditions often fail to account for sleep architecture, circadian rhythms, and physiological states that directly influence glymphatic activity [100,101]. This represents a significant conceptual gap. Without incorporating glymphatic dynamics, current models may overestimate the role of Aβ overproduction while underrepresenting impaired clearance mechanisms. Integrating glymphatic assessment, through imaging modalities such as diffusion-based imaging or tracer-based approaches, into rodent models could provide a more accurate representation of disease progression and therapeutic response [102]. It is essential to acknowledge that the causal predominance of glymphatic dysfunction in AD remains contentious. Numerous studies indicate that glymphatic alterations in transgenic models seem to be a consequence of, rather than a precursor to, amyloid accumulation. For instance, AQP4 knockout experiments have produced variable effects on amyloid burden across diverse model systems, with some studies documenting exacerbated pathology while others report unchanged or even diminished pathology. This mismatch likely indicates model-specific variables, such as genetic background, age during assessment, and sleep disturbance methods [99,100,101]. We define glymphatic dysfunction as an emerging and potentially significant aspect of disease rather than a confirmed primary cause, and we suggest that future models incorporate standardised glymphatic evaluation in conjunction with rather than as a replacement for assessments of Aβ production and clearance.

### 4.2. CSVD: The Vascular Contribution to Neurodegeneration

CSVD is increasingly recognized as a major contributor to cognitive decline and dementia, often coexisting with AD pathology. CSVD encompasses a range of microvascular abnormalities, including endothelial dysfunction, BBB breakdown, impaired cerebral perfusion, and white matter damage [103]. The interaction between vascular dysfunction and neurodegeneration is complex and bidirectional. Chronic hypoperfusion can exacerbate Aβ accumulation and tau pathology, while neuroinflammation and oxidative stress further compromise vascular integrity. Moreover, CSVD-related features such as enlarged perivascular spaces are closely linked to impaired glymphatic function, highlighting an important vascular–clearance axis in AD [104,105].

However, most rodent models of AD inadequately capture these vascular components. Transgenic models primarily focus on neuronal pathology, with limited representation of microvascular changes. Similarly, toxin-induced and lesion-based models rarely incorporate chronic vascular insufficiency or BBB dysfunction as primary drivers of disease. This omission is particularly problematic given that sporadic AD, which constitutes the majority of cases, is strongly associated with vascular risk factors such as hypertension, diabetes, and aging. As a result, current models may fail to replicate the vascular contributions that are critical in human disease.

To improve translational relevance, there is a need for models that integrate vascular pathology, including chronic hypoperfusion models, endothelial dysfunction paradigms, and multimodal approaches combining CSVD with amyloid or tau pathology. Such models would better reflect the neurovascular unit as a central player in AD progression.

### 4.3. Microbiota–Gut–Brain Axis: A Neglected Systemic Regulator

The microbiota–gut–brain axis represents a bidirectional communication network linking the central nervous system with the gastrointestinal tract, immune system, and metabolic pathways. Growing evidence suggests that gut microbiota composition influences neuroinflammation, BBB integrity, and even Aβ deposition [106]. Dysbiosis, characterized by an imbalance in microbial populations, has been associated with increased production of proinflammatory cytokines, endotoxins such as LPS, and metabolic by-products that can influence brain function [31]. These factors can enter systemic circulation, disrupt the BBB, and trigger neuroinflammatory cascades that contribute to neurodegeneration.

Although this axis is gaining attention in AD research, it remains poorly integrated into most rodent models. Standard laboratory practices, including controlled diets, sterile environments, and the use of antibiotics, can significantly alter the gut microbiome, potentially confounding experimental outcomes [107,108]. The evidence supporting the causative function of gut microbiota in AD is not consistently homogenous. Research employing germ-free mouse models has demonstrated considerable variability in amyloid pathology outcomes: certain studies indicated that germ-free APP/PS1 mice displayed diminished amyloid accumulation, whereas later replication efforts utilising diverse model backgrounds and housing conditions yielded inconsistent findings, with some laboratories observing no notable differences. This heterogeneity likely indicates the microbiome’s sensitivity to environmental factors such as cage co-housing, dietary composition, and bedding that are seldom standardised across studies [107,108,109,110]. Moreover, microbiome-targeted therapies like probiotics and faecal microbiota transplantation have exhibited variable efficiency across AD models, highlighting the context-dependent nature of this relationship. The limitations of reproducibility do not diminish the significance of the gut–brain axis as a disease modulator; instead, they underscore the necessity for systematic microbiome characterisation and standardised environmental controls as essential methodological prerequisites for inquiry. Future rodent models must include microbiome analysis and regulated nutritional manipulations, utilising germ-free or humanised microbiota systems exclusively under meticulously standardised settings.

### 4.4. Integrative Perspective: Toward Multi-Dimensional Modelling

Collectively, glymphatic dysfunction, CSVD, and the microbiota–gut–brain axis represent interconnected systems that are largely absent from conventional rodent models of AD. These dimensions are not independent; rather, they interact dynamically. For example, vascular dysfunction can impair glymphatic clearance, while gut-derived inflammation can exacerbate both vascular and neurodegenerative processes. The exclusion of these factors reflects a broader limitation in current experimental approaches: the tendency to model AD as a purely neurocentric disorder. This reductionist perspective may contribute to the persistent failure of translating preclinical findings into effective clinical therapies.

Incorporating these missing dimensions into rodent models represents a critical step toward a more holistic and clinically relevant understanding of AD. Multi-hit models that integrate protein aggregation, vascular dysfunction, metabolic alterations, sleep disruption, and microbiota changes may better capture the complexity of sporadic AD and improve the predictive value of preclinical research. Ultimately, advancing AD research will require a paradigm shift from isolated pathway modelling to integrative, systems-level approaches that reflect the disease’s true multifactorial nature.

## 5. Therapeutic Implications for Drug Development in AD

Despite substantial advances in understanding AD pathophysiology, therapeutic development has been marked by repeated clinical failures. A striking paradox persists, whereby numerous pharmacological agents demonstrate robust efficacy in preclinical rodent models yet fail to produce meaningful clinical benefit in humans. This disconnect highlights fundamental limitations not only in drug design but also in the experimental models that guide therapeutic discovery. Currently approved pharmacological treatments for AD are largely symptomatic, targeting neurotransmitter imbalances rather than underlying disease mechanisms. Acetylcholinesterase (AChE) inhibitors (donepezil, galantamine, rivastigmine) and the NMDA receptor antagonist memantine provide modest and transient improvements in cognition and behaviour. However, these agents do not halt neurodegeneration or prevent disease progression. The continued reliance on symptomatic therapies reflects the limited success of disease-modifying strategies and underscores the complexity of AD as a multifactorial disorder.

### 5.1. Disease-Modifying Therapies and Translational Failure: Amyloid Reduction Without Clinical Translation

The development of disease-modifying therapies for AD has been predominantly guided by the amyloid cascade hypothesis, resulting in the emergence of monoclonal antibodies such as aducanumab, lecanemab, and donanemab. These agents have demonstrated a clear ability to reduce amyloid burden in the brain [111]; however, their clinical impact remains modest and, in many cases, controversial. This disconnect highlights a fundamental issue in AD therapeutics, wherein amyloid reduction does not necessarily translate into meaningful cognitive improvement, particularly in patients with established or late-stage disease [112]. In addition, these therapies are associated with safety concerns, including amyloid-related imaging abnormalities (ARIA), which further complicate their clinical applicability [111,112].

The limited clinical success of anti-amyloid therapies is closely intertwined with the broader issue of translational failure in AD drug development. A major contributing factor is the reliance on rodent models that are inherently biased toward amyloid overproduction. These models are typically engineered to overexpress mutant forms of amyloid precursor protein, leading to accelerated and exaggerated amyloid pathology. While this design facilitates the evaluation of amyloid-targeting interventions, it does not accurately reflect the multifactorial and sporadic nature of AD in humans.

More broadly, existing rodent models capture only selected aspects of AD pathology, most commonly amyloid accumulation or tau aggregation, while failing to incorporate other critical contributors such as vascular dysfunction, glymphatic impairment, metabolic disturbances, and systemic inflammation. This reductionist framework creates a skewed therapeutic landscape in which amyloid-targeting strategies are disproportionately prioritized. Furthermore, treatments in preclinical studies are often administered during early or even pre-symptomatic stages of disease, whereas clinical interventions typically occur after significant neurodegeneration has already taken place. The absence of key comorbidities, including aging, cerebrovascular disease, and metabolic syndrome, further limits the translational relevance of these models [113]. Additionally, the rapid and genetically driven progression observed in rodent systems contrasts sharply with the slow, heterogeneous course of AD in humans.

As a result of these limitations, therapeutic agents that demonstrate robust efficacy in preclinical settings frequently fail to produce comparable benefits in clinical trials. This persistent gap underscores the need to move beyond reductionist, amyloid-centric approaches and toward more integrative models that better capture the complexity of AD. The magnitude of this translational failure is noteworthy. In numerous therapeutic domains, rodent models exhibit a positive predictive value for clinical efficacy in just 30–50% of cases, with this ratio being significantly lower in AD. A pivotal examination of clinical trials for AD from 1998 to 2017 revealed 146 ineffective therapeutic agents, resulting in an overall clinical failure rate of around 99.6% for disease-modifying medications [114]. The notable attrition rate far exceeds the failure rates in oncology (about 95%) and cardiovascular disease (around 80–85%), underscoring the unique translational challenges in modelling AD. The mechanical rationale for these failures provides insights that extend beyond just aggregate statistics. Semagacestat, a γ-secretase inhibitor that significantly diminished Aβ production in APP/PS1 and 5xFAD transgenic mice, paradoxically impaired cognitive function in a Phase III trial, likely due to the non-selective inhibition of Notch signaling, a consequence not anticipated by amyloid-centric mouse models that fail to replicate the complete spectrum of γ-secretase substrates [115]. Bapineuzumab, a monoclonal anti-Aβ antibody, demonstrated significant reductions in plaques and cognitive improvements in various transgenic mice models; however, it failed to exhibit any substantial impact on cognition or functional results in two extensive Phase III trials involving over 2000 patients. Solanezumab, which exhibited cognitive benefits in PDAPP and Tg2576 mice, similarly did not succeed in the Expedition I and II trials including people with mild to moderate AD [116]. The tau aggregation inhibitor TRx0237 (LMTM) demonstrated efficacy in the rTg4510 tau model but failed to meet primary endpoints in two Phase III trials [117]. The amyloid-related imaging abnormalities (ARIA) observed with the recent approval of monoclonal antibodies like lecanemab and aducanumab, which were largely unforeseen based on transgenic mouse models, highlight the insufficiency of current models to accurately reflect human cerebrovascular vulnerability. Collectively, these examples illustrate that the preclinical achievements in transgenic rodents reflect the model’s biology rather than the pathophysiology of human sporadic AD. Table 3 summarizes currently approved drugs and emerging therapeutic candidates for AD, along with their mechanisms of action and developmental status. Notably, the majority of therapies are either symptomatic or target single pathological pathways, reinforcing the need for more integrative approaches.

Clinical status reflects regulatory approval or trial stage at the time of writing and may vary across regions. Regulatory approval status is current as of March 2026 and may vary by country or region. Readers are encouraged to consult the relevant regulatory agencies for the most up-to-date information. Key limitations summarize major concerns reported in clinical studies, including efficacy, safety, and scope of therapeutic action. Development status reflects the most recent publicly available clinical trial phase or outcome. “Trials terminated” indicates discontinuation due to lack of efficacy or safety concerns. “Trials completed” refers to studies that have concluded but may not have met primary endpoints. “Ongoing trials” indicate active clinical evaluation. “Early-stage” refers to preclinical or Phase I/II investigations. AChE, acetylcholinesterase; NMDA, N-methyl-D-aspartate; Aβ, amyloid-beta; FDA, Food and Drug Administration; ARIA, amyloid-related imaging abnormalities; AD, Alzheimer’s disease.

### 5.2. Expanding the Therapeutic Landscape: Beyond Amyloid

Recent therapeutic strategies have increasingly shifted beyond the traditional amyloid-centric framework to target a broader spectrum of pathological processes implicated in AD. These include neuroinflammation, tau aggregation, synaptic dysfunction, mitochondrial impairment, vascular pathology, and metabolic dysregulation. This diversification reflects a growing recognition that AD is not driven by a single pathogenic pathway, but rather by a complex interplay of interconnected biological systems. Among these emerging approaches, modulation of neuroinflammation has gained significant attention, with therapies targeting microglial activation and cytokine signalling pathways [137]. Similarly, tau-directed therapies, including aggregation inhibitors, kinase inhibitors, and immunotherapies, aim to address neurofibrillary pathology more directly associated with cognitive decline [136]. In parallel, interventions targeting synaptic health and mitochondrial function seek to preserve neuronal integrity and energy metabolism [138].

Notably, the microbiota–gut–brain axis has emerged as a promising therapeutic target, with agents such as oligomannate demonstrating the potential to modulate systemic inflammation and indirectly influence neurodegenerative processes [124]. Additionally, strategies aimed at improving vascular function and enhancing glymphatic clearance are being explored to facilitate the removal of neurotoxic proteins and restore brain homeostasis [139]. Despite these advances, most non-amyloid therapies remain in early developmental stages, and many have yet to demonstrate consistent or robust clinical efficacy. This highlights a critical limitation: even when targeting alternative pathways, current therapeutic strategies often remain reductionist, focusing on isolated mechanisms rather than the integrated nature of disease progression.

Consequently, there is an increasing need to transition toward multi-dimensional therapeutic frameworks that simultaneously address multiple pathological domains. Such approaches may include combination therapies, multi-target drugs, and personalized treatment strategies tailored to individual disease profiles. Importantly, the success of these strategies will depend on the development of next-generation preclinical models that more accurately reflect the systemic and heterogeneous nature of AD, incorporating factors such as aging, vascular dysfunction, metabolic imbalance, and microbiota interactions. Ultimately, expanding the therapeutic landscape beyond amyloid is a necessary but insufficient step. Bridging the gap between experimental promise and clinical success will require not only broader target selection but also a fundamental shift toward systems-based thinking in both drug development and disease modelling.

## 6. Future Directions: Toward Next-Generation AD Modelling and Therapeutics

**Figure 5:**
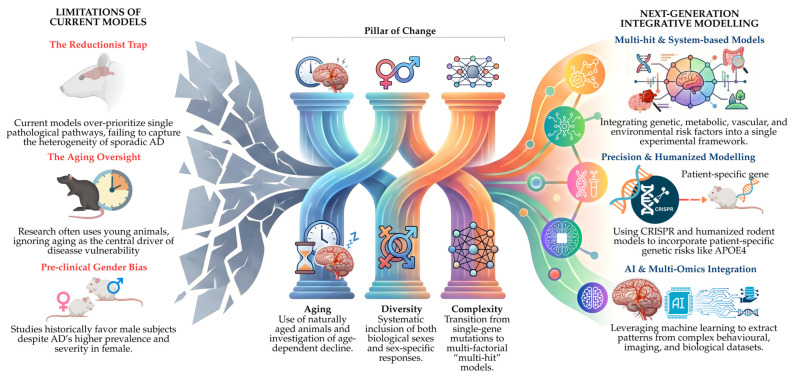
Bridging the translational gap in Alzheimer’s disease (AD) research. This infographic highlights the shift from traditional reductionist approaches toward next-generation integrative modelling to better capture the complexity of AD. Current limitations include an over-reliance on single-pathway models (e.g., amyloid-centric), inadequate consideration of aging as the primary risk factor, and a historical bias toward male subjects despite higher prevalence and distinct manifestations in women. To address these gaps, future research should prioritize aging-relevant models, the inclusion of both sexes, and multi-factorial disease complexity. Next-generation strategies emphasize multi-hit and systems-based models that integrate genetic, metabolic, vascular, and environmental influences, alongside precision approaches such as CRISPR and humanized models to reflect patient-specific risks (e.g., APOE4). The integration of artificial intelligence and multi-omics further enables comprehensive analysis of complex datasets, advancing translational relevance.

The persistent discrepancy between robust preclinical efficacy and repeated clinical failure in AD necessitates a fundamental re-evaluation of current experimental paradigms. Future progress in the field will depend on abandoning reductionist frameworks that prioritize single pathological pathways and instead adopting integrative, systems-level approaches that more accurately reflect the multifactorial nature of sporadic AD. Such a transition is essential not only for improving mechanistic understanding but also for enhancing the translational validity of preclinical findings (Figure 5).

### 6.1. Development of Concrete, Operationalised Criteria and Systems-Based Models

A critical limitation of existing rodent models lies in their reliance on familial AD mutations, which disproportionately emphasize amyloid overproduction and fail to capture the heterogeneity of sporadic disease. Given that sporadic AD arises from the convergence of multiple interacting risk factors, future models must incorporate combinatorial pathological drivers rather than isolated genetic alterations. The integration of genetic susceptibility, metabolic dysfunction, vascular impairment, neuroinflammatory processes, and environmental influences is essential for reproducing the complex disease milieu observed in humans.

A minimum-criteria framework for next-generation AD rodent models is needed. This framework is structured into three categories according to the extent of investigation and translational objectives (Table 4):

Tier 1—Fundamental requirements (pertinent to all novel AD rodent models): (i) Age stratification: studies focusing on chronic or progressive diseases must utilise animals aged ≥ 12 months at the commencement of the study. (ii) Baseline microbiota characterisation: gut microbiota composition must be analysed at both the beginning and conclusion using 16S rRNA sequencing or a comparable method. (iii) Sleep/circadian monitoring: a minimum of one 72-h actigraphy or EEG recording session must occur during the experimental phase. (iv) Vascular integrity assessment: at least one validated evaluation of blood–brain barrier permeability (e.g., Evans Blue extravasation, in vivo two-photon imaging, or MRI-based perivascular space quantification) is required.

Tier 2—Integrative requirements (for studies explicitly modelling sporadic Alzheimer’s Disease): (i) A minimum of two systemic risk-factor domains must be integrated (e.g., metabolic challenge [high-fat diet or intracerebroventricular streptozotocin] combined with chronic low-grade neuroinflammation [repeated low-dose lipopolysaccharide]). (ii) Glymphatic function must be evaluated using at least one validated method (DTI-ALPS index, tracer-based cerebrospinal fluid injection, or AQP4 immunofluorescence with polarisation quantification).

Tier 3—Translational requirements (for models designed to facilitate IND-enabling studies or clinical trial design): (i) Inclusion of the APOE4 knock-in allele. (ii) Sex-balanced cohorts with sex-stratified primary analysis. (iii) Multi-omics profiling (minimum: plasma proteomics + 16S rRNA microbiome). (iv) Advanced in vivo neuroimaging (structural MRI + FDG-PET or equivalent).

Practical and ethical considerations: Naturally aged rodent cohorts (≥18 months for mice; ≥24 months for rats) necessitate significantly extended living durations, with per-animal expenses projected to be 3–5 times greater than those of conventional experimental animals, according to published colony cost estimates. The implications of the 3Rs concept encompass the necessity for Refinement (improved welfare monitoring in elderly animals with comorbidity risks) and the conflict between Reduction (lower cohort sizes) and the requirements for statistical power. The consortium-based ageing colony sharing, typified by the NIA Aged Rodent Colony program, serves as an effective cost-reduction technique, and we advocate for the implementation of this model in the field. Multi-hit models that incorporate many mutations or stressors generate phenotypic complexity, necessitating larger cohorts for resolution and increasing the ethical burden. It is essential to explicitly recognise these real-world limits in the study design and a stepwise deployment of this tiered framework—initiating with Tier 1 for all new studies is advised as a practical starting point.

**Table 4:** Proposed minimum criteria by model tier.

Tier	Scope	Minimum Requirements	Cost/Ethical Considerations
Tier 1	All new AD rodent models	Age ≥ 12 mo; baseline microbiota; sleep monitoring; BBB assessment	Moderate; feasible with standard facilities
Tier 2	Sporadic AD modelling	Tier 1 + ≥2 systemic risk domains combined; glymphatic assessment	High; requires multi-stressor coordination
Tier 3	IND-enabling/clinical trial-informing	Tier 2 + APOE4 KI; sex-balanced; multi-omics; advanced neuroimaging	Very high; recommend consortium colony sharing

### 6.2. Incorporation of Aging as a Central Biological Driver

Despite being the most significant risk factor for AD, aging remains inadequately represented in most experimental models. The widespread use of young animals or accelerated disease paradigms fails to capture the progressive physiological decline associated with aging, including alterations in immune function, vascular integrity, and metabolic regulation [140]. This directly addresses the limitation identified in Section 3.1 that transgenic models rely on accelerated, non-physiological disease progression. Future research must prioritize the incorporation of aging as a central determinant of disease vulnerability. This requires the use of naturally aged animals and the systematic investigation of age-dependent processes such as impaired glymphatic clearance, chronic low-grade inflammation, and cerebrovascular dysfunction [141]. Aging does not act in isolation but interacts synergistically with other pathological mechanisms, amplifying susceptibility to neurodegeneration. Failure to account for these interactions may lead to an overestimation of therapeutic efficacy and contribute significantly to the translational gap observed in AD research.

### 6.3. Precision Modelling: Genetic and Sex-Specific Approaches

The heterogeneity of AD necessitates a shift toward precision modelling strategies that account for individual variability in disease risk and progression. Genetic factors, particularly the apolipoprotein E ε4 (APOE4) allele, play a central role in modulating multiple aspects of AD pathology, including amyloid accumulation, tau aggregation, neuroinflammation, and vascular dysfunction [142]. The CRISPR/Cas9-mediated APOE4 knock-in directly resolves the construct validity issue (Section 3.1) associated with reliance on familial AD mutations by incorporating genetically prominent sporadic AD risk into established transgenic models. Likewise, the replacement of humanised tau isoforms using CRISPR rectifies the tau splicing disparity in 4R/3R isoform ratios between rodents and humans, a particular mechanistic deficiency highlighted in the comparative validity evaluation (Table 3). Genetic humanisation alone is inadequate and must be integrated with the systemic prerequisites defined in Tier 2–3 above.

Equally important is the systematic consideration of biological sex as a determinant of disease susceptibility and progression. Epidemiological evidence consistently demonstrates a higher prevalence and severity of AD in women [143], yet preclinical studies have historically been biased toward male subjects. This directly relates to the sex-specific metabolic differences outlined in Section 3.3.1 (HFD models), wherein females and males exhibit divergent inflammatory and metabolic response patterns. Future research should implement sex-stratified primary analyses instead of considering sex as an exploratory variable. Moreover, the connection between sex and APOE4 constitutes a critical focus: females possessing APOE4 have significantly heightened AD risk, and models that incorporate this interaction may uncover mechanistically unique treatment targets.

### 6.4. Integration of Artificial Intelligence (AI) and Multi-Omics Approaches

Artificial intelligence (AI) and machine learning cannot replace biological accuracy: an AI model applied to a 5xFAD cohort devoid of glymphatic evaluation would not retrieve the absent biological aspect. The role of AI is analytical rather than ontological. AI addresses limitations in three distinct yet complementary ways to experimental model enhancements: (1) it integrates multi-parametric datasets from models that encompass glymphatic, vascular, and microbiota measurements, facilitating the identification of biomarker signatures reflective of these dimensions, even with incomplete datasets; (2) it enables cross-model comparisons to discern which preclinical findings are replicated across model systems, thereby highlighting model-specific artifacts such as transcriptomic profiles associated with APP overexpression that are absent in knock-in models that may elucidate failed clinical translation; and (3) it diminishes false-positive rates in drug screening by identifying confounding variables (e.g., cage microbiome composition, circadian phase during behavioural testing) that are infrequently controlled in traditional studies. When utilised in this manner, AI is most effective when integrated with next-generation models that already encompass the biological aspects; it enhances biological signals rather than compensating for their absence [144,145].

### 6.5. Toward Translationally Relevant and Humanized Models

Advances in genetic engineering technologies, particularly CRISPR/Cas9, have enabled the development of humanized rodent models that incorporate clinically relevant genetic variants with high precision [146]. Such models offer the potential to bridge the gap between experimental systems and human disease by more accurately replicating the molecular and cellular context of AD. However, genetic humanization alone is insufficient to capture the full complexity of the disease.

Future efforts must extend beyond genetic manipulation to include the integration of physiological and systemic factors that are critical to disease progression. This includes the incorporation of BBB dynamics, glymphatic function, microbiota composition, and systemic metabolic interactions. Additionally, the combination of rodent models with complementary platforms, such as human-induced pluripotent stem cell-derived organoids and advanced in vivo imaging techniques, may provide a more comprehensive and translationally relevant framework for studying AD pathogenesis.

### 6.6. Bridging the Translational Gap: A Conceptual Shift

Ultimately, overcoming the persistent failure of AD therapeutics requires a fundamental conceptual shift in how the disease is modelled and understood. Rather than viewing AD as a series of discrete pathological events, it must be conceptualized as a systems-level disorder arising from the dynamic interplay of neuronal, vascular, metabolic, and peripheral processes. This perspective necessitates the development of experimental frameworks that integrate these dimensions into cohesive models of disease. Such an approach has the potential to enhance the biological relevance of preclinical studies and to improve the likelihood of successful translation into clinical therapies. By aligning experimental design with the complexity of human disease, future research can move beyond the limitations of current models and establish a more reliable foundation for therapeutic innovation in AD. Collectively, these directions emphasize that future success in AD therapeutics will depend not only on novel drug targets but on the development of models that faithfully recapitulate the multidimensional nature of the disease.

## 7. Conclusions

Rodent models have been indispensable in advancing our understanding of AD, providing critical insights into Aβ deposition, tau pathology, synaptic dysfunction, and neuroinflammation. Transgenic models have enabled detailed mechanistic studies and supported preclinical therapeutic development, while chemically and metabolically induced models have broadened the experimental landscape by capturing aspects of sporadic AD, including insulin resistance, oxidative stress, and neuroinflammation. Despite these advances, a significant translational gap persists. The high failure rate of AD therapeutics in clinical trials highlights a fundamental limitation: most rodent models recapitulate only isolated features of the disease, often reflecting rare familial mutations rather than the multifactorial nature of sporadic AD. Consequently, therapies that show promise in preclinical studies frequently fail to demonstrate meaningful clinical benefit. This review emphasizes that AD is not solely a neurocentric disorder but a multidimensional, systems-level disease. Underrepresented factors, including glymphatic dysfunction, CSVD, metabolic imbalance, and the microbiota–gut–brain axis, play crucial roles in disease progression but are inadequately modelled. We have proposed a tiered minimum-criteria framework (Section 6.1) that converts this diagnostic observation into a definitive operational roadmap, delineating Tier 1 requirements for all new AD rodent studies and advancing to Tier 3 requirements for IND-enabling investigations. The most suitable model class is for specific experimental enquiries, addressing the perceived contradiction between criticisms of transgenic and toxin-based models: both are beneficial within their respective contexts, yet neither is adequate independently for translational drug development. Emerging dimensions glymphatic dysfunction and the microbiota–gut–brain axis are identified as potentially significant although methodologically diverse, necessitating standardised assessment methodologies prior to the conclusive establishment of their causative involvement. Future progress will depend on next-generation models that integrate aging, genetic susceptibility, vascular pathology, and systemic influences into unified frameworks. The incorporation of multi-omics, neuroimaging, and artificial intelligence may further enhance translational relevance. Ultimately, advancing AD therapeutics requires a shift toward integrative, multidimensional models that better reflect human disease complexity, thereby bridging the gap between experimental success and clinical efficacy.

## Figures and Tables

**Table 1:** Dose-dependent effects of intracerebroventricular (ICV) streptozotocin (STZ) in rodent models of Alzheimer’s disease (AD).

STZ Dose (ICV)	Administration Protocol	Key Phenotypic Outcomes	Experimental Use	Translational Relevance	Key Limitations
≤0.5–1 mg/kg [64]	Single or split dose (bilateral ventricles)	Mild cognitive impairment, early insulin signalling disruption, subtle oxidative stress	Early-stage AD modelling; neuroprotection studies	Mimics prodromal/metabolic dysfunction phase	Weak pathology; variability across studies
~1–2 mg/kg [65,66]	Commonly 2 × injections (Day 1 & Day 3)	Moderate cognitive deficits, neuroinflammation, cholinergic dysfunction, Aβ accumulation	Widely used sporadic AD model	Best approximation of sporadic AD features (brain insulin resistance)	Rapid onset; partial representation of pathology
≥3 mg/kg [63]	Single or repeated high-dose injection	Severe neurodegeneration, marked oxidative stress, neuronal loss	Acute neurotoxicity studies	Poor, resembles toxic injury rather than AD	Non-specific toxicity; low clinical relevance
Fixed dose (e.g., 3 mg/kg total ICV) [67]	Bilateral ICV injection (often 1.5 mg/kg per ventricle)	Consistent cognitive impairment, mitochondrial dysfunction	Standardized protocols in the literature	Moderate reproducibility across labs	Still lacks tau pathology and slow progression

**Table 2:** Summary of rodent models used in Alzheimer’s disease (AD) research.

Model Category	Model Name	Key Features/Outcomes	Major Strengths	Key Limitations	Best Use	Not Suitable For
Transgenic Rat Models	UKUR25, McGill-R-Thy1-APP	hAPP expression; intracellular Aβ accumulation without plaque formation	Early-stage amyloid pathology; useful for mechanistic studies	Lack of extracellular plaques; incomplete AD phenotype	Mechanistic studies of early intracellular Aβ accumulation	Studies requiring extracellular plaque pathology
	Tg478/Tg1116	Development of amyloid plaques (late onset)	The first rat models showing plaque formation	Late pathology onset; limited tau pathology	Long-term studies of amyloid plaque evolution	Studies requiring early-onset pathology or tau involvement
	PSAPP Rat	APP + PSEN mutation; accelerated plaque formation	Improved amyloid pathology modelling	Off-target effects (e.g., hypertension, renal pathology)	Accelerated amyloid pathology studies	Studies sensitive to cardiovascular or renal confounds
	TgF344-AD	Both amyloid plaques and tau-like pathology, as well as cognitive decline	One of the most comprehensive rat AD models	Tau pathology not fully human-like; limited reproducibility across labs	Combined amyloid and tau pathology and behavioural studies	Sporadic AD research without genetic drivers
	Human Tau Rat Models	Expression of mutant human tau; NFT formation	Useful for tau-specific mechanisms	Lack of amyloid component; incomplete disease spectrum	Tau-driven neurodegeneration mechanisms	Studies requiring amyloid co-pathology
Transgenic Mouse Models	APP/PS1	Early amyloid plaque deposition; neuroinflammation; cognitive deficits	Widely used; reproducible; suitable for anti-amyloid studies	Minimal tau pathology; amyloid-centric bias	Anti-amyloid drug screening and reproducibility studies	Sporadic AD, vascular contributions, and aging-related AD research
	5xFAD	Rapid and aggressive Aβ pathology; neuronal loss; early cognitive impairment	Fast disease progression; ideal for drug screening	Overexpression artifacts; poor modelling of sporadic AD and tau pathology	Rapid-throughput drug screening for Aβ-targeted therapies	Sporadic AD and tau-pathology research
	3xTg-AD	Both amyloid and tau pathology; age-dependent progression	Models of the interaction between Aβ and tau	Slow progression; variability; limited neuronal loss	Studying amyloid-tau interaction over disease progression	Vascular or metabolic risk-factor research
	Tau Models (P301S, rTg4510)	Robust tau pathology, NFTs, neurodegeneration	Excellent for studying tau-driven neurotoxicity	No amyloid component; limited relevance to full AD	Tau-driven neurotoxicity and NFT formation studies	Studies requiring amyloid pathology
Lesion-Induced Models	ICV Streptozotocin (STZ)	Insulin resistance, neuroinflammation, and cognitive decline	Mimics sporadic AD metabolic dysfunction	Artificial induction; lacks full amyloid/tau pathology	Modelling brain insulin resistance and sporadic AD metabolic features	Studies requiring genetically driven amyloid/tau pathology
	Aβ Injection Model	Rapid amyloid deposition; synaptic dysfunction; memory deficits	Fast and controllable; useful for Aβ toxicity studies	Non-physiological Aβ levels; no NFT formation	Short-term Aβ toxicity and synaptic dysfunction studies	Chronic or NFT-related disease modelling
Toxin-Induced Models	Scopolamine Model	Cholinergic blockade; memory impairment; oxidative stress	Useful for cognitive and drug screening studies	Transient effects; no true neurodegeneration	Rapid cognitive/drug screening assays	Studies requiring sustained neurodegeneration
	Aluminium Chloride (AlCl_3_)	Promotes Aβ aggregation, tau phosphorylation, and neuroinflammation	Models’ environmental neurotoxicity	Controversial relevance to human AD; non-specific toxicity	Studying environmental/metal-induced neurotoxic mechanisms	Studies requiring high translational relevance to human AD
	D-galactose Model	Oxidative stress, aging-like phenotype, and cognitive decline	Models aging-related processes	Does not replicate core AD hallmarks (Aβ/tau)	Studying oxidative stress and aging-related cognitive decline	Studies requiring core Aβ/tau pathology
Metabolic Models	High-Fat Diet/Obesity	Insulin resistance, inflammation, and cognitive impairment	Links AD with metabolic syndrome	Variable reproducibility; weak plaque/tangle pathology	Investigating metabolic syndrome-AD links	Studies requiring robust plaque/tangle pathology
Inflammation-Based Models	LPS-Induced Neuroinflammation	Microglial activation, cytokine release, and neuroinflammation	Models’ immune contribution to AD	Acute inflammation; lacks chronic disease progression	Studying acute neuroinflammatory mechanisms	Modelling chronic, progressive neuroinflammation
Surgical Models	Cholinergic Lesion Models	Loss of basal forebrain cholinergic neurons; cognitive deficits	Models’ cholinergic hypothesis	Does not replicate full AD pathology	Testing cholinergic-targeted therapeutic interventions	Studies requiring full AD pathological spectrum

Aβ: Amyloid-beta; AD: Alzheimer’s disease; APP: Amyloid precursor protein; PSEN: Presenilin; NFT: Neurofibrillary tangle; ICV: Intracerebroventricular; STZ: Streptozotocin; LPS: Lipopolysaccharide; AlCl_3_: Aluminium chloride.

**Table 3:** Summary of currently approved pharmacological and ongoing experimental therapies for Alzheimer’s disease (AD), including their mechanisms of action, clinical status, and key limitations.

**Ref.**	**Active Molecule**	**Mechanism of Action**	**Clinical Status**	**Key Limitation**
[118,119]	Donepezil	AChE inhibitor	Approved	Symptomatic only; no disease modification
[120,121]	Galantamine	AChE inhibitor	Approved	Limited efficacy in later stages
[122]	Rivastigmine	AChE inhibitor	Approved	Gastrointestinal side effects
[121,123]	Memantine	NMDA receptor antagonist	Approved	Modest benefit in severe AD only
[124]	Oligomannate	Anti-inflammatory; gut microbiota modulation	Conditional approval (China)	Limited global validation
[125]	Aducanumab	Anti-Aβ monoclonal antibody	FDA accelerated approval (2021); discontinued by manufacturer (2024)	Controversial efficacy; ARIA risk not currently marketed
[125,126]	Lecanemab	Anti-Aβ monoclonal antibody	Approved (selected regions)	Modest clinical benefit
[127]	Donanemab	Anti-Aβ monoclonal antibody	FDA Approved (2024)	Safety concerns; limited long-term data
[128]	Brexpiprazole	Serotonin/dopamine modulator	Approved (agitation in AD)	Does not target core pathology
**Experimental** **Therapies**				
**Ref.**	**Active Molecule**	**Mechanism of Action**	**Development Status**	**Key Limitation**
[129]	Semagacestat	γ-secretase inhibitor	Trials terminated	Worsened cognition; toxicity
[130]	ALZ-801	Inhibits Aβ oligomer formation	Ongoing trials	Limited clinical validation
[131]	Varoglutamstat	Glutaminyl cyclase inhibitor	Ongoing trials	Uncertain long-term efficacy
[132]	Tideglusib	Tau kinase inhibitor	Trials completed	Limited efficacy
[133]	TRx0237	Tau aggregation inhibitor	Trials completed	Failed primary endpoints
[134]	ALZT-OP1	Enhances Aβ clearance	Trials completed	Inconclusive outcomes
[135]	Masitinib	Neuroimmune modulator	Early-stage	Limited clinical data
[136]	AADvac1	Anti-tau vaccine	Trials completed	Modest immunogenicity

## Data Availability

No new data were created or analyzed in this study. Data sharing is not applicable to this article.

## Abbreviations

The following abbreviations are used in this manuscript:

AChE	Acetylcholinesteras
AD	Alzheimer’s disease
AI	Artificial intelligence
AlCl_3_	Aluminium chloride
APOE	Apolipoprotein E
APP	Amyloid precursor protein
APPswe	Swedish mutant of human amyloid precursor protein
AQP4	Aquaporin-4
ARIA	Amyloid-related imaging abnormalities
ATP	Adenosine triphosphate
Aβ	Amyloid-beta
BBB	Blood–brain barrier
Ca^2+^	Calcium ion
CAT	Catalase
COX-2	Cyclooxygenase-2
CSF	Cerebrospinal fluid
CSVD	Cerebral small vessel disease
Cu	Copper
D-gal	D-galactose
FDDA	Food and Drug Administration
Fe	Iron
GPx	Glutathione peroxidase
hAPP	Human amyloid precursor protein
HFD	High-fat diet
ICV	Intracerebroventricular
IL	Interleukin
iNOS	Inducible nitric oxide synthase
ISF	Interstitial fluid
LPS	Lipopolysaccharides
mAChRs	Muscarinic acetylcholine receptors
NFTs	Neurofibrillary tangles
NF-κB	Nuclear factor kappa B
NLRP3	NLR family pyrin domain containing 3
NMDARs	N-methyl-D-aspartate receptors
PS1-ΔE9	ΔE9 variant of human presenilin 1
PSEN	Presenilins
ROS	Reactive oxygen species
SOD	Superoxide dismutase
STZ	Streptozotocin
TLR4	Toll-like receptor 4
TNF-α	Tumour necrosis factor-alpha
UPS	Ubiquitin–proteasome system
WHO	World health organization
Zn	Zinc
